# Microplastic accumulation in riverbed sediment via hyporheic exchange from headwaters to mainstems

**DOI:** 10.1126/sciadv.abi9305

**Published:** 2022-01-12

**Authors:** Jennifer D. Drummond, Uwe Schneidewind, Angang Li, Timothy J. Hoellein, Stefan Krause, Aaron I. Packman

**Affiliations:** 1 School of Geography, Earth and Environmental Sciences, University of Birmingham, Edgbaston, Birmingham, UK.; 2 Department of Civil and Environmental Engineering, Northwestern University, Evanston, IL, USA.; 3 Department of Biology, Loyola University Chicago, Loyola, IL, USA.; 4 LEHNA–Laboratoire d’ecologie des hydrosystemes naturels et anthropises, Villeurbanne, France.

## Abstract

In rivers, small and lightweight microplastics are transported downstream, but they are also found frequently in riverbed sediment, demonstrating long-term retention. To better understand microplastic dynamics in global rivers from headwaters to mainstems, we developed a model that includes hyporheic exchange processes, i.e., transport between surface water and riverbed sediment, where microplastic retention is facilitated. Our simulations indicate that the longest microplastic residence times occur in headwaters, the most abundant stream classification. In headwaters, residence times averaged 5 hours/km but increased to 7 years/km during low-flow conditions. Long-term accumulation for all stream classifications averaged ~5% of microplastic inputs per river kilometer. Our estimates isolated the impact of hyporheic exchange processes, which are known to influence dynamics of naturally occurring particles in streams, but rarely applied to microplastics. The identified mechanisms and time scales for small and lightweight microplastic accumulation in riverbed sediment reveal that these often-unaccounted components are likely a pollution legacy that is crucial to include in global assessments.

## Teaser

Bidirectional flow between surface water and sediment leads to high accumulation of small and lightweight microplastics in rivers.

## INTRODUCTION

Plastic production and the amount of plastic waste are growing at an exponential rate. Plastics are pervasive to all ecosystems globally and will persist over long time scales (*1*, *2*). Rivers are considered a major source of plastic to oceans (*3*) with a considerable proportion being classified as microplastics (MPs), defined recently as particles <1 mm (*4*) but more widely as all particles <5 mm. However, studies on accumulation, export, and residence times of plastic particles within riverine systems have only recently emerged (*5*, *6*). MPs enter freshwater systems as both primary plastic objects (i.e., designed to be small) and secondary particles (i.e., fragments derived from breakdown of larger plastic objects) (*1*, *3*). Although small MPs (≤100 μm) are often not measured, recent estimates suggest that they can be ~6 × 10^8^ times more abundant than larger size classes in freshwater sediment (*7*) and account for 11.6 to 21.1 million tons of plastic waste suspended in the top 200 m of the Atlantic Ocean (*8*).

MPs are polluting freshwater ecosystems from both point and nonpoint sources. Point sources include wastewater discharges, combined sewer overflows, runoff from sealed surfaces, and accidental spills (*9*, *10*). MPs enter the domestic wastewater stream via synthetic textile fragments and fibers in washing machine effluent, beads in soaps or scrubbers, fragments, and other particles in runoff, leading to a wide variety of MP types (i.e., size, shape, and density) within freshwater ecosystems (*11*). The smaller the plastic, the higher the probability of passing through any removal system designed to stop their transport into the environment. For instance, municipal wastewater treatment plants (WWTPs) filter between 96 and 99.9% of MP in influent water but still release 1 to 100 MP/liter in treated effluent, with a higher likelihood that smaller MPs will pass through the filtration system (*9*, *12*). Nonpoint sources of MPs include atmospheric deposition, road runoff, littering, and sludges of WWTPs distributed to agricultural soils that can be eventually transported to freshwaters (*13*). In addition, up to 80% of wastewater globally is untreated, yielding much higher nonpoint source MP loadings to freshwater systems (*14*).

In previous studies, small MPs ≤ 100 μm were assumed to transport downstream with minimal interaction with riverbed sediment because of their low gravitational settling velocity (*15*, *16*). However, the transport of MPs ≤ 100 μm, similar to that of other small naturally occurring particles, is strongly influenced by turbulent flow and groundwater–surface water interactions driven by streambed topographic effects that lead to considerable hyporheic exchange, the bidirectional flow between surface water and sediment in rivers (*7*, *17*). Particle retention by hyporheic exchange, which has been validated from decades of research and empirical evidence [e.g., see review paper (*18*)], traps a wide range of particles in the river benthos, including lightweight plastics that might otherwise be expected to float. Prior studies have demonstrated that accumulation of small MPs in riverbeds is ongoing, but hyporheic exchange processes have not yet been incorporated into hydrologic models, which quantify pathways for small particle retention. New models that estimate the time scales of hyporheic MP dynamics (i.e., deposition, retention, and resuspension) are critical, yet missing components needed to quantify MP abundance in riverine systems and the associated environmental and public health risks (*19*). Here, we quantify MPs residence times and accumulation for streams classified as headwaters to mainstems deduced from global river data, isolating the impact of the often unaccounted for hyporheic exchange processes. We focus on the less well-studied yet more abundant small MPs (≤100 μm), which have a higher surface-to-volume ratio and therefore higher likelihood to serve as a vector of harmful pathogens and are more readily available for uptake by organisms, potentially degrading ecosystem health (*13*, *20*).

### How are small MPs transported in rivers?

**Fig. 1.**
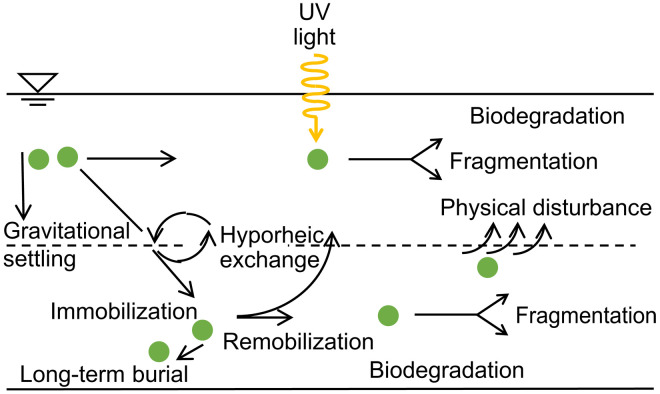
Processes that control MP accumulation in rivers. Both gravitational settling and hyporheic exchange transport MPs into riverbed sediment, followed by either long-term burial, biodegradation and fragmentation, or remobilization to the water column.

MP deposition into underlying sediment has long been assumed to be driven solely by heteroaggregation with other particles and gravitational settling (*15*, *16*), but hydrodynamics exerts more complex forces on particle transport and retention in flowing waters. Although settling occurs within rivers and should be especially considered for larger and denser particles, fine particles ≤ 100 μm are mainly transported into riverbeds by hyporheic exchange flow (*17*, *18*, *21*). Hyporheic exchange is driven by pressure variations at the riverbed surface and turbulent momentum transfer into porewater (Fig. 1). Rates of fine particle deposition by hyporheic exchange flow can be up to 180 times greater than those for gravitational settling (*22*). For MPs, the ratio of hyporheic exchange rate to gravitational settling rate can increase to over 100,000 (*17*), indicating that hyporheic exchange is a critical mechanism for MP transport from rivers into bed sediment, especially for smaller size fractions. Previous empirical measurements showed that MP retention follows similar patterns to naturally occurring allochthonous particles, including low-density organic particles, which accumulate in riverbed sediment (*23*).

Exchanged particles deposit or become immobilized within riverbed sediment by a variety of mechanisms including granular filtration, settling in porewater, and retention in benthic and hyporheic biofilms (*18*, *21*). While well studied for naturally occurring particles, the implications of these processes for MP retention in riverbed sediment have not been considered to date. Once deposited in the riverbed, MPs may be remobilized to the water column, chemically degraded, fragmented into smaller pieces, ingested by benthic and hyporheic fauna, or buried and stored for long periods of time (*13*, *24*). High stream flows commonly remobilize deposited particles when the critical shear stress of the bed is exceeded and bed sediment is remobilized (*25*), but high flows can also move MP particles deeper into bed sediment and thus enhance long-term plastic burial in riverbeds (*26*). The primary mechanism for plastic degradation in the water column is ultraviolet (UV) light, but this is greatly restricted in sediment. Biological degradation of plastic polymers also occurs in both the surface water and sediment, but biodegradation rates have been estimated to be slow (*13*). In addition, fragmentation reduces the particle size, increasing the abundance of smaller MPs over time. Overall, MP concentrations in riverbed sediment are expected to be greater than in surface waters due to substantial accumulation of MPs in sediment via hyporheic exchange and the expected limitations of plastic degradation in the hyporheic environment. Over long time scales, some fraction of the MPs that accumulate in riverbed sediment during low-flow periods are remobilized during floods (*25*). Accumulated MP may eventually propagate to the ocean, or to inland depositional areas, through a series of flood events that occur over geologic time scales (*27*). However, neither the average storage time scale of MPs nor the fraction of particles that are remobilized versus buried long-term is currently known.

Models that only consider gravitational settling, even with biofilm colonization, predict that smaller MPs (≤100 μm) and larger buoyant MPs will not accumulate within riverbed sediment (*16*) unless heteroaggregation is considered (*28*). Yet, these assumptions do not coincide with observations of large amounts of small and low-density plastics in riverbed sediment worldwide (*5*, *6*). This discrepancy between observed and predicted accumulation of lightweight MP particles in streambed environments suggests that additional processes are responsible for MP deposition and retention in sediment, beyond those included in existing models. We aim to quantify the accumulation and residence times of MPs from hyporheic exchange processes, which will especially affect the fate of small MPs ≤ 100 μm as shown by previous fine particle and MPs experimental evidence (*18*, *26*).

## RESULTS

### Model development and validation

We here use a mobile-immobile model framework (see Materials and Methods) to quantify MP accumulation in riverbed sediment and residence times of exported particles for a wide range of hydrologic conditions from headwaters to mainstems in global river networks (i.e., headwaters, small creeks, large creeks, small rivers, medium rivers, and mainstems). For this purpose, we adapted a model that has been widely used to simulate transport and accumulation of particulate organic matter, fine inorganic particles, and microbial cells in streams and rivers (*21*, *26*). The model considers exchange from the water column to riverbeds (i.e., due to hyporheic exchange), immobilization, and remobilization, as well as downstream transport of MPs (Fig. 1). Deposition and immobilization processes generally dominate the behavior of fine particles within riverbed sediment (*18*, *21*), leading to long-term accumulation.

We first use our model to interpret a field dataset (*7*) and assess whether observed stream hydrologic conditions and ranges of accumulation time scales (i.e., 1 to 6 months) could account for the high number of small MPs (10,000 to 50,000 #/kg dry weight, 20 to 50 μm) measured in streambed sediment downstream of a WWTP effluent (see Materials and Methods). This dataset is unique as it measures MPs as small as 20 μm, and buoyant polymers (polypropylene and polyethylene) were the largest fraction of the plastics found in the size range of 20 to 50 μm. Therefore, a model that incorporates hyporheic exchange, and not only gravitational settling, was needed to explain observations of such small and buoyant particles in riverbed sediment only ~150 m downstream of their point source. We found multiple input parameter sets, representing a realistic range of hydrologic conditions and MP properties that can account for the numbers of small MPs measured in the streambed sediment (Table 1 and Materials and Methods). We assumed that MPs were only sourced from the WWTP although we acknowledge other sources are expected in an urban setting. Therefore, this study represents a conservative input scenario and demonstrates that the model can represent how small, lightweight MPs accumulate in streambed sediment when hyporheic exchange processes are considered.

**Table 1.** Case study parameter inputs and outputs for model validation. Descriptions of input parameters were based on published values, scaling relationships using known parameters, or assumptions between solute and fine particle transport with equations and references identified. For other unknown parameters, variability was incorporated with Monte Carlo simulations (*N* = 10,000 parameter runs) with upper and lower limits listed in the table (see Materials and Methods). Bolded variables are model inputs parameters.

**Parameter** **description**	**Unit**	**Parameter range or** **estimate, equation,** **or reference**
Discharge of WWTP effluent	liter/s	30–460 (*42*, *43*)
MP in WWTP effluent	#/liter	54 (*12*)
**MP input**, MPs input to stream	#/s	1000 to 25,000
*Q*, average stream flow	m^3^/s	2.1 (*42*, *43*)
*B*, stream width	m	4.4, calculated from *Q* (*44*–*46*)
*h*, average stream depth	m	0.44, calculated from Q (*44*–*46*)
***v***, velocity	m/s	1.1, *Q*/(*Bh*)
*S*, slope	–	0.004
***D***, dispersion	m^2^/s	3.7 (*47*)
**β_S_**, power law slope for solute immobile zone residence time distribution	–	0.2–1
**Λ_P_**, Exchange from the water column to immobile zone	1/s	6.3 × 10^−4^, Eq. 3 (*39*, *40*)
**Λ_IP_**, particle exchange rate within the immobile zone	1/s	Set to Λ_P_ (*37*)
**β_IP_**, power law slope for the particle residence time distribution within the immobile zone	–	Set to β_S_/2 (*37*)

### Predictions of MP accumulation and residence times in global rivers

**Fig. 2.**
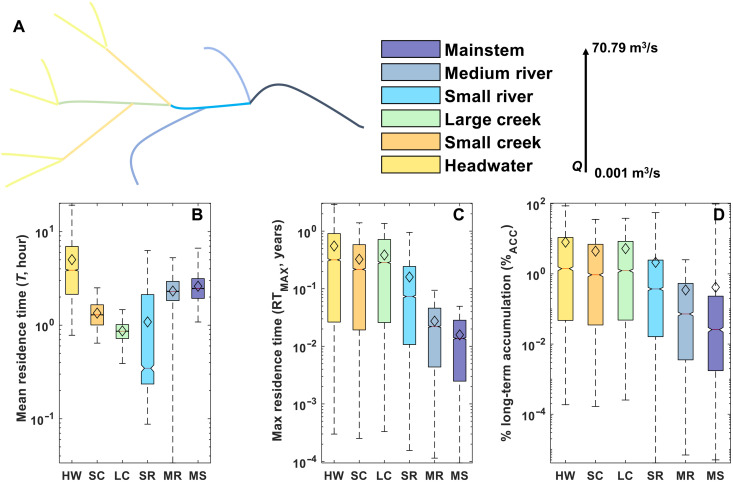
Retention times and accumulation of MPs in riverbed sediment per kilometer in headwaters to mainstems for the range in average annual streamflow conditions. Schematic of stream classification by discharge (**A**). MP (**B**) mean residence times ($\bar{T}$), (**C**) maximum residence time (RT_MAX_) for the particles that transport downstream and are not retained, and (**D**) percentage of long-term accumulation (i.e., >317 years) in riverbed sediment (%_ACC_) per kilometer by stream classification of headwaters to mainstems (Table 2). The black diamond is the mean value for each stream classification. HW, headwater; SC, small creek; LC, large creek; SR, small river; MR, medium river; MS, mainstem.

We quantified residence times of small lightweight MP particles in global rivers, focusing on all polymers in the size range of ≤100 μm or any size if buoyant, along with the long-term accumulation rate of MPs per kilometer (Table 2, Fig. 2, and see Material and Methods). We assessed MP transport and deposition for hydrologic conditions for streams classified as headwaters to mainstems (*29*, *30*) with mobile-immobile model simulations (see Materials and Methods). The stream classification system relates to the Strahler orders 1 to 6 for headwaters to mainstems, respectively, but with the classes based on discharge and geomorphic transitions instead of only on position in the stream network (Table 2) (*29*, *30*). River discharge is variable over time, and “average streamflow” represents an annual composite of low-flow (baseflow) and high-flow (stormflow) conditions. We first evaluate each stream classification considering the range in average stream annual flow conditions and then assess baseflow conditions. To assess the effects of variation in hydrologic conditions for each stream classification, we ran Monte Carlo simulations (*N* = 10,000) varying the range in values for hydrologic input parameters (Table 2). For average annual streamflow conditions, we found that mean and maximum MP residence times were greatest in headwater streams (Fig. 2, A and B). While the maximum residence time consistently decreased from headwaters to mainstem streams, the mean residence time was shortest for large creeks, with hydrologic conditions defined in Table 2. The variation in mean residence times from headwaters to mainstems reflects the persistent increase in stream velocity and width (Table 2). This occurs because of the impact of stream velocity on hyporheic exchange. These patterns resulted in a wide range of long-term accumulation rates and time scales, with highest retention in headwaters, the most abundant stream classification (Fig. 2C and Table 2).

**Table 2.** Hydrological conditions for global rivers classified as headwaters to mainstems used to calculate input parameters and model outputs of residence times of exported particles and accumulation of MPs in riverbed sediment. Description of input parameters were based on published values, scaling relationships using known parameters, or assumptions between solute and fine particle transport with equations and references identified. For other unknown parameters, variability was incorporated with Monte Carlo simulations (*N* = 10,000 parameter runs) with upper and lower limits listed in the table (see Materials and Methods). Bolded variables are model inputs parameters. HW, headwater; SC, small creek; LC, large creek; SR, small river; MR, medium river; MS, mainstem.

	***Q* (m^3^/s)**	***B* (m)**	***H* (m)**	***S***	***v* (m/s)**	***D* (m^2^/s)**	**Λ_P_ (1/s)**	**β_S_**	**Λ_IP_ (1/s)**	**β_IP_**	**Count**	**Length (km)**
	(*30*)	(*29*)	(*44*–*46*)	(*30*)		(*47*)	(*39*, *40*)		(*37*)	(*37*)	(*30*)	(*30*)
HW	0–0.057	0.8–3	Calc. from rel. to *Q*	0–0.001	*Q*/(*Bh*)	Eq. 6		0.2–1			1481646	2947149
SC	0.057–0.283	3–4.4		0.001–0.005			Eq. 4				542257	1139800
LC	0.283–1.133	4.4–13.8		0.005–0.02			3.5 × 10^−6^–9.9 × 10^−4^		Λ_P_	β_S_/2	261629	502085
SR	1.133–5.663	13.8–72.5		0.02–0.04							169653	289972
MR	5.663–22.65	72.5–170.8		0.04–0.1							84392	132600
MS	22.65–70.79	170.8–532.9		0.1–1							41830	61607

We assessed watershed-scale retention and downstream efflux of MPs by combining information on residence times, long-term accumulation rates, and flow paths for each stream classification under average annual streamflow (i.e., 100% baseflow condition). Our projections indicate that MPs that enter headwater streams accumulate in the sediment at a rate of 8% per kilometer on average. For mainstem rivers, MPs that enter the river are retained at a rate of 3% per kilometer (Fig. 2). The other remaining ~92 to 97% per kilometer of MPs are exported another kilometer further downstream in the river network, with a mean residence time of 5 and 0.1 hours/km, and a much longer maximum residence time of 0.6 and 0.2 years/km in headwaters and mainstems, respectively (Fig. 2). However, the probability for MPs to reach the oceans before accumulation decreases with distance from the source input location to the ocean, with each incremental kilometer leading to increased accumulation and residence times along the way. For example, we can consider a 10 km reach between a MP source and the freshwater-marine interface. In this case, a 5% per kilometer MP long-term accumulation rate causes a large fraction of the MPs (~50%) to be retained in riverbed sediment before reaching the ocean. Furthermore, with a mean residence time of ~3 hours/km and a maximum residence time of 0.3 years/km, the exported MPs will be retained within the river for 30 hours on average and 3 years maximum. This retention time provides the opportunity for abiotic and biological processes (i.e., biofilm colonization and consumption by animals) to alter the particle properties and fate before reaching the marine environment.

**Fig. 3.**
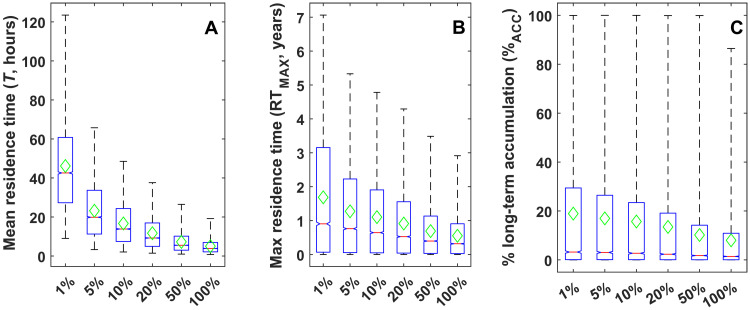
MP retention and accumulation estimates for varying baseflow conditions in headwater streams. Baseflow was assessed as 1, 5, 10, 20, 50, and 100% of the annual stream flow with model outputs for (**A**) mean residence times (*T*), (**B**) maximum residence time (RT_MAX_) for the particles that transport downstream and are not retained, and (**C**) percentage of long-term accumulation in riverbed sediment (%_ACC_) per kilometer. The green diamond is the mean value for each stream classification.

Since the most frequent river discharge is only a fraction of the average annual streamflow (*31*), we repeated the simulations under baseflow conditions assuming 1, 5, 10, 20, and 50% of the average annual streamflow. We ran these simulations for headwater streams, which are the most globally abundant stream classification and the greatest fraction of overall river length (*29*, *30*) (Table 2) and have the greatest MP accumulation rates and residence times under annual average flow conditions (Fig. 2). Residence time of exported MPs increased as stream flow decreased. For example, at 1% average annual streamflow, the average MPs residence time was 46 hours/km, 9 times greater than for average annual conditions, (Fig. 3A), and the maximum was 1.7 years/km, 2.8 times greater than average annual conditions (Fig. 3B). Under these conditions, MP accumulation also increased to 2.4 times the average annual conditions to 19% per kilometer on average (Fig. 3).

## DISCUSSION

This study presents the first assessment of MP accumulation and residence times within freshwater systems from headwaters to mainstems from hyporheic exchange processes, focusing on the hard-to-measure yet abundant size fraction ≤ 100 μm. We focused on the influence of hyporheic exchange processes on MP fate in streams, which is rarely studied. Thus, our results represent minimum estimates as other processes such as biofilm colonization, heteroaggregation, and settling by gravity are only expected to increase deposition and retention (*23*, *32*). Our analyses indicated the importance of riverbed sediment as a long-term sink for MPs (~3 to 8% per kilometer), while the remaining 92 to 97% of the inputs are stored in the riverbed sediment for as long as ~0.2 to 0.6 years/km before downstream export. Further, our results show that MPs are preferentially retained in headwater systems under low-flow conditions, yielding accumulation rates up to 19% per kilometer and residence times up to 1.7 years/km. MP accumulation dependence on flow conditions is particularly relevant given projections of future increases in frequency, magnitude and duration of meteorological extremes, and associated high- and low-flow events.

Our findings contribute a first estimate for budgets of MPs ≤ 100 μm within rivers and their storage and redistribution between the continents and the oceans. Future studies that characterize the spatiotemporal distribution of MP accumulation in streams, combined with models that incorporate varying flow conditions and overbank flow, will improve predictions of MP accumulation and export. Our results demonstrate the potential importance of headwaters retaining MPs. The high accumulation of small MPs in headwaters is likely due to the combined effects of high hyporheic exchange rates due to shallow turbulent waters and the longer residence times within the sediment as compared to large rivers. These findings are relevant to in situ dynamics as small streams can receive WWTP effluent (*33*) and associated MPs, along with MPs from a suite of other sources such as agricultural and septic drainage, stormwater inputs, and aerial deposition (*24*). These advances will enable identification of hotspots of MP accumulation, such as headwaters, which will facilitate ecological impact assessment and mitigation measures including cleanup of the most polluted locations by knowing where increased controls on MP inputs can make the largest differences to global distributions.

The synthesis of our model results highlights that lightweight and small MPs accumulate substantially in riverbed sediments. This emphasizes the importance of global budgets to consider freshwaters as an important reservoir of long-term storage of MPs. A crucial difference between rivers and other aquatic systems such as lakes, reservoirs, or wetlands is that the advective water flow and turbulent exchange that characterizes hyporheic exchange processes delivers large numbers of MPs, especially the smaller size fractions, into riverbed sediment (*7*, *17*). We provide the first estimates of MP accumulation in riverbed sediment for global rivers through hyporheic exchange processes under the influence of dynamic hydrologic conditions from headwaters to mainstems. Our results are based on readily available information on river hydrologic and geomorphic conditions and represent a rather conservative estimate of MP deposition. However, the influence of groundwater inputs was not considered, which can both suppress or increase hyporheic exchange and associated deposition of fine particles (*18*, *34*, *35*). Even higher accumulation rates and residence times can be expected for most rivers since our model estimates do not incorporate the effects of biofilm colonization, including benthic algal mats and hyporheic bacterial biofilms, which increases attachment and retention of MPs in riverbeds.

Long-term MP accumulation and persistence are expected to vary with polymer type and environmental factors that influence particle properties. Although MPs are highly persistent in riverbed sediment, UV radiation and physical, chemical, and biological activities can degrade deposited particles. These processes not only increase the particle numbers via fragmentation but also transform some fraction of plastic to inorganic gases (*36*). Many MPs will become incorporated into complex organic-inorganic aggregates in rivers and estuaries, but this process has not been quantified to date because natural heteroaggregation is still difficult to measure. Transport, retention, and plastic breakdown all vary with polymer type (*17*, *23*). Fine particle immobilization in bed sediment also depends on their size, density, and surface chemistry. The current model provides a general basis to assess the effects of hyporheic exchange and deposition on MP accumulation in sediment with a focus especially on the size fraction ≤ 100 μm. Density and size effects, including fragmentation and aggregation, can be included in the mobile-immobile model framework as more detailed data become available. We therefore recommend that future studies incorporate polymer-specific transport, retention, and breakdown characteristics to improve predictions of MP fate.

Beyond the hydrologic processes considered here, floods deliver high loads of MPs into the river from terrestrial sources while simultaneously exporting remobilized MPs from the bed sediment further downstream or overbank, where they deposit onto floodplains. High-flow events not only have the capacity to remobilize a portion of MPs from riverbed sediment (*25*) but also potentially drive the remaining particles into deeper and less-mobile regions (*26*), leading to long-term burial. An improved understanding of the net effect of accumulation and remobilization during floods is needed to improve predictions for MPs storage in river systems and the rate and time scale of MP export to the oceans. We recommend future field measurements and modeling approaches to consider the effect of discrete events on the redistribution of plastic particles throughout the river continuum.

## MATERIALS AND METHODS

### Experimental design

We estimate in-stream residence times and long-term accumulation of MPs in global rivers, classified from headwaters to mainstems. We start by validating a mobile immobile model framework to field data that measures the smaller size fraction ≤ 100 μm (*7*) and then expand the analysis to assess streams classified from headwaters to mainstems.

### Mobile-immobile model

#### Model overview and equations

The mobile-immobile model simulates the transport and retention of MPs in streams and has been previously applied to studies of fine particle transport (*21*, *26*, *37*). The mobile-immobile model is governed by advection and dispersion processes convolved with a memory function to represent storage in the system (*21*, *37*)

$$\frac{\partial C(x,t)}{\partial t}=\int_{0}^{t}M(t-t\prime )[-v\frac{\partial C(x,t\prime )}{\partial x}+D\frac{\partial^{2}C(x,t\prime )}{\partial x^{2}}]\mathrm{dt}\prime$$ (1)

where *C* [*M L*^−3^] is in-stream MP concentration, *t* [*T*] is the elapsed time, *t*′ [T] is a dummy time variable, *x* is downstream distance [*L*], *M*(*t*) [*T*^−1^] is the memory function, and *v* [*L T*^−1^] and *D* [*L*^2^ *T*^−1^] are the velocity and dispersion coefficient in the mobile zone (i.e., water column). The memory function (Eq. 2) is dependent on the overall residence time distribution of MPs in the stream, ψ_P_(*t*) [*T*^−1^]. The Laplace transform of the memory function *M*(*t*) is

$$\tilde{M}(u)=u\bar{t}\frac{{\tilde{\psi }}_{P}(u)}{1-{\tilde{\psi }}_{P}(u)}$$ (2)

where *u* [*T*^−1^] is the Laplace variable and $\bar{t}$ is the average travel time in the reach, defined as the stream reach length divided by the mean water velocity (*v*). ψ_P_ (*t*)[*T*^−1^] is defined by the residence time distribution in the mobile zone (i.e., water column), ψ_0_(*t*) [*T*^−1^], the rate of exchange from the water column to the immobile zone, Λ_P_ (*t*) [T^−1^], and the residence time distribution of MPs in the immobile zone, φ_P_(*t*) [*T*^−1^]

$${\tilde{\psi }}_{P}(u)={\tilde{\psi }}_{0}[u+\Lambda_{P}-\Lambda_{P}{\tilde{\varphi }}_{P}(u)]$$ (3)

The immobile zone is defined as encompassing all stream storage areas, and in our study, we focus on the riverbed sediment. We assume that small MPs (≤100 μm) are transported in the stream water column identically to solutes owing to their small settling velocities (*38*), shown to have an exponential residence time distribution in the water column set to ψ_0_(*t*) = *e*^−*t*^ (*18*, *21*). For the same reason, we assume that delivery of MPs to the immobile zone is controlled purely by hyporheic exchange processes and that gravitational settling is negligible. Therefore, the model provides a lower-case scenario for MP transport from the water column to riverbed sediment that considers only hyporheic exchange processes, as this rate could be enhanced by gravitational settling. For a specific river reach, the hyporheic exchange rate defined as the rate of exchange from the water column to the immobile zone, can be calculated experimentally via a solute tracer injection study (*21*). This has been the assumption incorporated into this model for many studies, including field MP injection studies, and has been an accurate estimate of exchange of small and lightweight MPs into the sediment (*17*, *26*, *37*). In need of a more generic approach that encompasses a variety of river hydraulic and channel characteristics, we estimated the hyporheic exchange rate, i.e., the exchange from the water column to the immobile zone from a scaling relationship developed by dimensional analysis with stream tracer data from 35 streams (*39*, *40*) as

$$\begin{array}{c} \frac{1}{\Lambda_{P}}=20.595(\frac{1}{0.097f^{0.42}}){\left(\frac{v}{u_{*}}\right)}^{-1.4625}{\left(\frac{B}{h}\right)}^{0.6639}\\ {\left(\frac{\mathrm{vL}}{D}\right)}^{0.3232}{S_{i}}^{1.9132}(\frac{h}{u_{*}}) \end{array}$$ (4)

where *h* is stream depth, *B* is stream width, *f* is the Darcy-Weisbach friction factor calculated as *f* = 8*ghS*/*v*^2^, u_*_ is the shear velocity calculated as u_*_ = (*ghS*)^0.5^ with *S* as the slope and *g* as the gravitational acceleration,*S_i_* is reach sinuosity ratio, and *L* is reach length. A representative *S_i_* for global rivers was estimated as 1.2 (*41*).

Once in the immobile zone, MPs can either transport with solutes directly back to the water column via porewater hyporheic flow paths or immobilize (Fig. 1). Therefore, the residence time distribution of MPs, φ_P_, describes both the delay in downstream transport that results from particles entering the immobile zone and following solute transport paths and further immobilization-remobilization within the immobile zone (e.g., from reversible deposition, filtration, and attachment)

$${\tilde{\varphi }}_{P}(u)={\tilde{\varphi }}_{S}[u+\Lambda_{\text{IP}}-\Lambda_{\text{IP}}{\tilde{\varphi }}_{\text{IP}}(u)]$$ (5)

where φ_S_ is the solute residence time distribution in the immobile zone, Λ_IP_ is the rate of MP particle immobilization within the immobile zone, and φ_IP_ is the particle residence time distribution in the immobile region. Both solute and particle residence time distributions in the immobile zone have been identified previously as power law with φ_S_ (*t*) ~*t*^−(1 + β_S_^^)^ and φ_IP_(*t*) ~ *t*^−(1 + β_IP_^^)^, where β_S_ and β_IP_ are power law slopes between 0 and 1 (*18*, *21*). A smaller value of β_S_ or β_IP_ indicates more time spent in the immobile zone, which reflects slower hyporheic transport and hence riverbed sediment properties that extend the residence time distribution of solutes and fine particles.

#### Model validation

To validate the mobile-immobile model, we compared model outputs to measured streambed sediment MPs in the smaller size range of 20 to 50 μm, ~150 m downstream of a WWTP measured in low–summer flow conditions (May and August 2017) in the Roter Main River (*7*). Briefly, sampling involved extracting a freeze core down to approximately 60 cm from a natural riffle structure by hammering a stainless-steel pipe into the sediments, filling with a mixture of dry ice and ethanol, and removing the pipe as soon as the surrounding sediments froze (~20 min). The core was investigated for MP particles in the size range of 20 to 500 μm using focal plane array–based micro–Fourier transform infrared spectroscopy (*7*). MP measurements reported in # liter^−1^ were converted to # m^−2^ by using the reported 10-cm depth (*h*) and sediment composition (70% coarse to medium sand and 30% medium to fine gravel) to estimate a sediment density of 1600 kg m^−3^. A conservative estimate of the number of MPs sourced from a WWTP effluent is based on data in a Danish WWTP with 99.3% removal efficiency, as 54 #/liter (*12*). Combined with the range in discharge of the WWTP from 360 to 460 liter/s (*42*, *43*), MP inputs (#/s) to the stream can vary between 1000 and 25,000 #/s. As the quantity of MPs within riverbed sediment represent months to years of accumulation, a comparison was made between the original observations by (*7*) (i.e., >1 × 10^6^ #/m^2^) and our model estimates after 1, 2, 3, and 6 months of MP continuous inputs.

#### Model inputs

The model assumes steady state conditions and MPs can be added as a pulse or continuous input. Input parameters are shown in Table 1. Stream depth *h* (m) and width *B* (m) were determined by the bank full relationships between *Q*, *B*, and *h* of 230 rivers worldwide (*44*–*46*). Velocity *v* was calculated from the median discharge *Q*, (m^3^/s) reported from April to July (*42*, *43*) as *Q*/(*Bh*). The dispersion coefficient *D* (m^2^/s) was calculated as (*47*)

D={2.0(Bh)1.5hu*,0<Bh<100(7.428+1.775(Bh)0.62(vu*)0.572)(vu*)hv,100<Bh<200 (6)

There is no available relationship to predict the other three unknown model parameters Λ_IP_, β_S_, and β_IP_. When particle data are not available, a reasonable estimate of particle immobilization can be drawn from solute parameters as was shown in previous field studies of both MPs and other fine particles (*37*). Specifically, particle interactions in the immobile zone can be estimated as Λ_IP_ = Λ_P_, assuming that increased exchange from the water column to the immobile zone will lead to a linear increase in particle immobilization within the immobile zone. Last, β_IP_ = β_S_/2 provides a reasonable estimate (*37*), indicating that the increased particle interactions compared to solutes will lead to increased immobilization and delay the remobilization back to the water column with a heavier (less steep) slope, therefore only leaving β_S_ as unknown.

We performed several computational experiments (*N* = 10,000) with simulations and parameter sets [only varying β_S_ and MP input (#/s)] by sampling the parameter space using a Latin Hypercube approach (*37*). β_S_ ranged from 0.2 to 1 (lower limit set by the limit within the model) and MP input ranged from 1000 to 25,000 #/s, assuming that the main source of MPs to the stream was from the WWTP.

#### Model outputs

Model outputs include MP particle counts at two known distances downstream (site 1 = 50 m and site 2 = 200 m) in the surface water for each model time step. These counts were integrated using the trapezoidal method to determine a total number of MPs that passed by site 1 and site 2 divided by the stream reach area using the average width to calculate an average number of MPs retained within the reach (#/m^2^) for each time duration (i.e., 1, 2, 3, and 6 months). We then compared the model outputs to measured plastics (>1 × 10^6^ #/m^2^) (*7*). As the time scale for accumulation increased, there was an increased number of input parameter sets that could have led to the measured MPs (7.4, 12.8, 69.7, and 71.4% of *N* = 10,000 input parameter sets for 1, 2, 3, and 6 months of accumulation, respectively).

#### Model predictions

Stream classification from headwaters to mainstems provides a wide range in hydrologic properties for global rivers (*29*, *30*). We varied average annual discharge (*Q*), stream width and slope using the ranges for each classification (Table 2). Following this, the remaining input parameters (Table 2) were estimated as in the model validation and run for *N* = 10,000 parameter sets for each classification type (headwaters, small creeks, large creeks, small river, medium river, and mainstem). We then assessed variations in baseflow conditions assuming 1, 5, 10, 20, and 50% of the annual stream discharge represented baseflow conditions, assessed for headwater streams only.

For each model run, we estimated three key parameters: mean residence time (*T*) and maximum residence time (RT_MAX_) of exported particles and the percentage of long-term accumulation of MPs (%_ACC_). All variables are provided per kilometer stream reach by comparing site 1 = 100 m to site 2 = 500 m for a model duration of 1 s to 1 × 10^10^ s (317 years). For an estimate of the mean residence time of exported particles, a pulse injection (60 s) of a 1-year input of MPs of 3.15 × 10^8^ particles was simulated to represent a low-input scenario (~10 MP/s). As an estimate of the short-term retention of MPs, we calculated the mean residence time of exported particles $T=\frac{\int (\mathrm{Ct})\mathrm{dt}}{\int \mathrm{Cdt}}$ and RT_MAX_ as the latest time one MP particle was detected at site 2. This value represents the timeframe for the short-term downstream transport of MPs, while the remaining MPs are assumed to stay immobilized for much longer than the model simulation (i.e., >317 years). This percentage of long-term MP accumulation (%_ACC_) was calculated as the ratio of the difference in integrated mass measured between site 2 and site 1 normalized by the site 1 integrated mass.

## References

[1] R. Geyer, J. Jambeck, K. Law, Production, use, and fate of all plastics ever made. Sci. Adv. 3, e1700782 (2017). 28776036 10.1126/sciadv.1700782 PMC5517107

[2] S. B. Borelle, J. Ringma, K. L. Law, C. C. Monnahan, L. Lebreton, A. McGivern, E. Murphy, J. Jambeck, G. H. Leonard, M. A. Hilleary, M. Eriksen, H. P. Possingham, H. De Frond, L. R. Gerber, B. Polidoro, A. Tahir, M. Bernard, N. Mallos, M. Barnes, C. M. Rochman, Predicted growth in plastic waste exceeds efforts to mitigate plastic pollution. Science 369, 1515–1518 (2020). 32943526 10.1126/science.aba3656

[3] J. R. Jambeck, R. Geyer, C. Wilcox, T. R. Siegler, M. Perryman, A. Andrady, R. Narayan, K. L. Law, Plastic waste inputs from land into the ocean. Science 347, 768–771 (2015). 25678662 10.1126/science.1260352

[4] N. B. Hartmann, T. Hüffer, R. C. Thompson, M. Hassellöv, A. Verschoor, A. E. Daugaard, S. Rist, T. Karlsson, N. Brennholt, M. Cole, M. P. Herrling, M. C. Hess, N. P. Ivleva, A. L. Lusher, M. Wagner, Are we speaking the same language? Recommendations for a definition and categorization framework for plastic debris. Environ. Sci. Technol. 53, 1039–1047 (2019). 30608663 10.1021/acs.est.8b05297

[5] S. Lambert, M. Wagner, in *Freshwater Microplastics*, M. Wagner, S. Lambert, Eds. (2017), pp. 1–24.

[6] A. Bellasi, G. Binda, A. Pozzi, S. Galafassi, P. Volta, R. Bettinetti, Microplastic contamination in freshwater environments: A review, focusing on interactions with sediments and benthic organisms. Environ. MDPI. 7, 30 (2020).

[7] S. Frei, S. Piehl, B. S. Gilfedder, M. G. J. Löder, J. Krutzke, L. Wilhelm, C. Laforsch, Occurrence of microplastics in the hyporheic zone of rivers. Sci. Rep. 9, 15256 (2019). 31649312 10.1038/s41598-019-51741-5 PMC6813303

[8] K. Pabortsava, R. S. Lampitt, High concentrations of plastic hidden beneath the surface of the Atlantic Ocean. Nat. Commun. 11, 4073 (2020). 32811835 10.1038/s41467-020-17932-9 PMC7434887

[9] K. Waldschläger, S. Lechthaler, G. Stauch, H. Schüttrumpf, The way of microplastic through the environment – Application of the source-pathway-receptor model (review). Sci. Total Environ. 713, 136584 (2020). 32019016 10.1016/j.scitotenv.2020.136584

[10] T. M. Karlsson, L. Arneborg, G. Broström, B. C. Almroth, L. Gipperth, M. Hassellöv, The unaccountability case of plastic pellet pollution. Mar. Pollut. Bull. 129, 52–60 (2018). 29680567 10.1016/j.marpolbul.2018.01.041

[11] X. Li, L. Chen, Q. Mei, B. Dong, X. Dai, G. Ding, E. Y. Zeng, Microplastics in sewage sludge from the wastewater treatment plants in China. Water Res. 142, 75–85 (2018). 29859394 10.1016/j.watres.2018.05.034

[12] M. Simon, N. van Alst, J. Vollertsen, Quantification of microplastic mass and removal rates at wastewater treatment plants applying focal plane array (FPA)-based Fourier transform infrared (FT-IR) imaging. Water Res. 142, 1–9 (2018). 29804032 10.1016/j.watres.2018.05.019

[13] S. Krause, V. Baranov, H. A. Nel, J. D. Drummond, A. Kukkola, T. Hoellein, G. H. Sambrook Smith, J. Lewandowski, B. Bonnet, A. I. Packman, J. Sadler, V. Inshyna, S. Allen, D. Allen, L. Simon, F. Mermillod-Blondin, I. Lynch, Gathering at the top? Environmental controls of microplastic uptake and biomagnification in freshwater food webs. Environ. Pollut. 268, 115750 (2021). 33172701 10.1016/j.envpol.2020.115750

[14] R. Connor, *The United Nations world water development report 2017: Wastewater: The untapped resource* (2017).

[15] M. Kooi, E. Besseling, C. Kroeze, A. P. van Wezel, A. A. Koelmans, in *Freshwater Microplastics*, M. Wagner, S. Lambert, Eds. (Springer, Berlin, 2018), pp. 125–152.

[16] L. Nizzetto, G. Bussi, M. N. Futter, D. Butterfield, P. G. Whitehead, A theoretical assessment of microplastic transport in river catchments and their retention by soils and river sediments. Environ Sci Process Impacts 18, 1050–1059 (2016). 27255969 10.1039/c6em00206d

[17] J. D. Drummond, H. A. Nel, A. I. Packman, S. Krause, Significance of hyporheic exchange for predicting microplastic fate in rivers. Environ. Sci. Technol. Lett. 7, 727–732 (2020).

[18] F. Boano, J. W. Harvey, A. Marion, A. I. Packman, R. Revelli, L. Ridolfi, A. Wörman, Hyporheic flow and transport processes. Rev. Geophys. 52, 603–679 (2014).

[19] K. Bucci, M. Tulio, C. M. Rochman, What is known and unknown about the effects of plastic pollution: A meta-analysis and systematic review. Ecol. Appl. 30, e02044 (2019). 10.1002/eap.2044 31758826

[20] E. Boelee, G. Geerling, B. van der Zaan, A. Blauw, A. D. Vethaak, Water and health: From environmental pressures to integrated responses. Acta Trop. 193, 217–226 (2019). 30857860 10.1016/j.actatropica.2019.03.011

[21] J. D. Drummond, A. F. Aubeneau, A. I. Packman, Stochastic modeling of fine particulate organic carbon dynamics in rivers. Water Resour. Res. 50, 4341–4356 (2014).

[22] K. R. Roche, J. D. Drummond, F. Boano, A. I. Packman, T. J. Battin, W. R. Hunter, Benthic biofilm controls on fine particle dynamics in streams. Water Resour. Res. 53, 222–236 (2017).

[23] T. J. Hoellein, A. J. Shogren, J. L. Tank, P. Risteca, J. J. Kelly, Microplastic deposition velocity in streams follows patterns for naturally occurring allochthonous particles. Sci. Rep. 9, 3740 (2019). 30842497 10.1038/s41598-019-40126-3 PMC6403300

[24] T. J. Hoellein, C. M. Rochman, The “plastic cycle”: A watershed-scale model of plastic pools and fluxes. Front. Ecol. Environ. 19, 176–183 (2021).

[25] R. Hurley, J. Woodward, J. J. Rothwell, Microplastic contamination of river beds significantly reduced by catchment-wide flooding. Nat. Geosci. 11, 251–257 (2018).

[26] J. D. Drummond, R. J. Davies-Colley, R. Stott, J. P. Sukias, J. W. Nagels, A. Sharp, A. I. Packman, Microbial transport, retention, and inactivation in streams: A combined experimental and stochastic modeling approach. Environ. Sci. Technol. 49, 7825–7833 (2015). 26039244 10.1021/acs.est.5b01414

[27] P. Regnier, P. Friedlingstein, P. Ciais, F. T. Mackenzie, N. Gruber, I. A. Janssens, G. G. Laruelle, R. Lauerwald, S. Luyssaert, A. J. Andersson, S. Arndt, C. Arnosti, A. V. Borges, A. W. Dale, A. Gallego-Sala, Y. Goddéris, N. Goossens, J. Hartmann, C. Heinze, T. Ilyina, F. Joos, D. E. Larowe, J. Leifeld, F. J. R. Meysman, G. Munhoven, P. A. Raymond, R. Spahni, P. Suntharalingam, M. Thullner, Anthropogenic perturbation of the carbon fluxes from land to ocean. Nat. Geosci. 6, 597–607 (2013).

[28] E. Besseling, J. T. K. Quik, M. Sun, A. A. Koelmans, Fate of nano- and microplastic in freshwater systems: A modeling study. Environ. Pollut. 220, 540–548 (2017). 27743792 10.1016/j.envpol.2016.10.001

[29] J. A. Downing, J. J. Cole, C. M. Duarte, J. J. Middelburg, J. M. Melack, Y. T. Prairie, P. Kortelainen, R. G. Striegl, W. H. McDowell, L. J. Tranvik, Global abundance and size distribution of streams and rivers. Inland Waters 2, 229–236 (2012).

[30] R. A. McManamay, C. R. Derolph, Data descriptor: A stream classification system for the conterminous United States. Sci. Data 6, 190017 (2019). 30747915 10.1038/sdata.2019.17 PMC6371895

[31] M. D. Dettinger, H. F. Diaz, Global characteristics of stream flow seasonality and variability. J. Hydrometeorol. 1, 289–310 (2000).

[32] C. D. Rummel, A. Jahnke, E. Gorokhova, D. Kühnel, M. Schmitt-Jansen, Impacts of biofilm formation on the fate and potential effects of microplastic in the aquatic environment. Environ. Sci. Technol. Lett. 4, 258–267 (2017).

[33] A. R. McCormick, T. J. Hoellein, M. G. London, J. Hittie, J. W. Scott, J. J. Kelly, Microplastic in surface waters of urban rivers: Concentration, sources, and associated bacterial assemblages. Ecosphere 7, e01556 (2016).

[34] A. Fox, A. I. Packman, F. Boano, C. B. Phillips, S. Arnon, Interactions between suspended kaolinite deposition and hyporheic exchange flux under losing and gaining flow conditions. Geophys. Res. Lett. 45, 4077–4085 (2018).

[35] N. Trauth, C. Schmidt, M. Vieweg, U. Maier, J. H. Fleckenstein, Hyporheic transport and biogeochemical reactions in pool-riffle systems under varying ambient groundwater flow conditions. J. Geophys. Res. Biogeosci. 119, 910–928 (2014).

[36] S.-J. Royer, S. Ferrón, S. T. Wilson, D. M. Karl, Production of methane and ethylene from plastic in the environmnent. PLOS ONE 13, e0200574 (2018). 30067755 10.1371/journal.pone.0200574 PMC6070199

[37] J. D. Drummond, N. Schmadel, C. Kelleher, A. I. Packman, A. Ward, Improving predictions of fine particle immobilization in streams geophysical research letters. Geophys. Res. Lett. 46, 13853–13861 (2019).

[38] S. Cook, H. L. Chan, S. Abolfathi, G. D. Bending, H. Schäfer, J. M. Pearson, Longitudinal dispersion of microplastics in aquatic flows using fluorometric techniques. Water Res. 170, 115337 (2020). 31830655 10.1016/j.watres.2019.115337

[39] T. S. Cheong, B. A. Younis, I. W. Seo, Estimation of key parameters in model for solute transport in rivers and streams. Water Resour. Manag. 21, 1165–1186 (2007).

[40] B. L. O’Connor, M. Hondzo, J. W. Harvey, Predictive modeling of transient storage and nutrient uptake: Implications for stream restoration. J. Hydraul. Eng. 136, 1018–1032 (2010).

[41] R. P. de Moraes Frasson, T. M. Pavelsky, M. A. Fonstad, M. T. Durand, G. H. Allen, G. Schumann, C. Lion, R. E. Beighley, X. Yang, Global relationships between river width, slope, catchment area, meander wavelength, sinuosity, and discharge. Geophys. Res. Lett. 46, 3252–3262 (2019).

[42] A. M. Becker, S. Gerstmann, H. Frank, Perfluorooctane surfactants in waste waters, the major source of river pollution. Chemosphere 72, 115–121 (2008). 18291438 10.1016/j.chemosphere.2008.01.009

[43] A. M. Becker, M. Suchan, S. Gerstmann, H. Frank, Perfluorooctanoic acid and perfluorooctane sulfonate released from a waste water treatment plant in Bavaria, Germany. Environ. Sci. Pollut. Res. 17, 1502–1507 (2010). 10.1007/s11356-010-0335-x 20419475

[44] C. Li, M. J. Czapiga, E. C. Eke, E. Viparelli, G. Parker, Variable Shields number model for river bankfull geometry: Bankfull shear velocity is viscosity-dependent but grain size-independent. J. Hydraul. Res. 53, 36–48 (2015).

[45] G. Parker, P. R. Wilcock, C. Paola, W. E. Dietrich, J. Pitlick, Physical basis for quasi-universal relations describing bankfull hydraulic geometry of single-thread gravel bed rivers. Case Rep. Med. 112, 1–21 (2007).

[46] G. V. Wilkerson, G. Parker, Physical basis for quasi-universal relationships describing bankfull hydraulic geometry of sand-bed rivers. J. Hydraul. Eng. 137, 739–753 (2010).

[47] Y. H. Zeng, W. X. Huai, Estimation of longitudinal dispersion coefficient in rivers. J. Hydro Environ. Res. 8, 2–8 (2014).

